# Brillouin Corrosion Expansion Sensors for Steel Reinforced Concrete Structures Using a Fiber Optic Coil Winding Method

**DOI:** 10.3390/s111110798

**Published:** 2011-11-16

**Authors:** Xuefeng Zhao, Peng Gong, Guofu Qiao, Jie Lu, Xingjun Lv, Jinping Ou

**Affiliations:** 1 School of Civil Engineering, Dalian University of Technology, Dalian 116024, China; E-Mails: reynold0510@126.com (P.G.); lujie719@163.com (J.L.); lvxingjun@163.com (X.L.); oujinping@dlut.edu.cn (J.O.); 2 School of Civil Engineering, Harbin Institute of Technology, Harbin 150090, China; E-Mail: qgf_forever@hit.edu.cn

**Keywords:** fiber optic, Brillouin sensor, corrosion sensor, fiber optic coil, steel reinforced concrete structure, structural health monitoring

## Abstract

In this paper, a novel kind of method to monitor corrosion expansion of steel rebars in steel reinforced concrete structures named fiber optic coil winding method is proposed, discussed and tested. It is based on the fiber optical Brillouin sensing technique. Firstly, a strain calibration experiment is designed and conducted to obtain the strain coefficient of single mode fiber optics. Results have shown that there is a good linear relationship between Brillouin frequency and applied strain. Then, three kinds of novel fiber optical Brillouin corrosion expansion sensors with different fiber optic coil winding packaging schemes are designed. Sensors were embedded into concrete specimens to monitor expansion strain caused by steel rebar corrosion, and their performance was studied in a designed electrochemical corrosion acceleration experiment. Experimental results have shown that expansion strain along the fiber optic coil winding area can be detected and measured by the three kinds of sensors with different measurement range during development the corrosion. With the assumption of uniform corrosion, diameters of corrosion steel rebars were obtained using calculated average strains. A maximum expansion strain of 6,738 με was monitored. Furthermore, the uniform corrosion analysis model was established and the evaluation formula to evaluate mass loss rate of steel rebar under a given corrosion rust expansion rate was derived. The research has shown that three kinds of Brillouin sensors can be used to monitor the steel rebar corrosion expansion of reinforced concrete structures with good sensitivity, accuracy and monitoring range, and can be applied to monitor different levels of corrosion. By means of this kind of monitoring technique, quantitative corrosion expansion monitoring can be carried out, with the virtues of long durability, real-time monitoring and quasi-distribution monitoring.

## 1. Introduction

**Figure 1.**
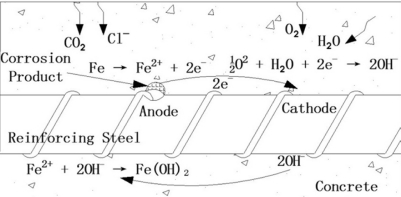
Schematic diagram of reinforcing steel corrosion in concrete as an electrochemical process.

Steel corrosion has become a major problem worldwide, especially for structures exposed to aggressive environments. This problem has reached alarming proportions in the past three decades, leading to very high repair costs, sometimes even above the initial construction cost, or in extreme situations, to the final collapse of the structures [1–4]. Figure 1 shows the schematic diagram of the corrosion process of the reinforcing steel rebar in concrete [5]. Concrete normally provides a high degree of protection to the reinforcing steel against corrosion, by virtue of the high alkalinity (pH > 13.5) of the pore solution. Under high alkalinity, steel rebar remains passivated. However, when sufficient chloride ions (from de-icing salts or from seawater) have penetrated to the steel rebar in concrete or as the pH value of the pore solution drops to low values due to the carbonation process, the protective film on the steel rebar surface is destroyed and the reinforcing steel is depassivated. Corrosion in the form of rust formation and loss in cross-section of the rebar in the presence of oxygen and water (humidity) then occur. The corrosion of steel in concrete is essentially an electrochemical process [6–8].

Although the corrosion mechanics of the reinforcing steel are well-known, finding out the corrosion status of the structure in service is very urgent in practical engineering structures. Over the past two decades, structural health monitoring (SHM) has gained worldwide acceptance as an economical way to obtain real-time data on the health, and subsequently the safety and serviceability of infrastructure systems [9]. Corrosion monitoring can provide much valuable information to SHM, the accurate monitoring of corrosion status of the steel rebar embedded in concrete in service life has been a technical challenge for a long time [10].

Recently, kinds of novel corrosion sensors have been applied in the health monitoring of large-scale civil structures such as buildings, dams, tunnels, river levees and other structures [11–13]. Among them, the fiber optical corrosion sensors are currently attracting considerable research interest in SHM owing to their distributed sensing ability, long sensing distance, high sensitivity, good durability, immunity to electromagnetic interference and other advantages [14,15]. Fuhr and Huston developed the fiber optical chloride detector, whose sensing mechanism relies on spectroscopic analysis of a chemical reaction of chloride and reagents [16]. Bennett designed a prototype optical fiber sensor for monitoring corrosion on large steel structures where the sensor works by pulling a multimode fiber into a tight bend and securing it with a “corrosion fuse” [17]. Li proposed a Fiber Optic Corrosion Sensor (FOCS) fabricated by electroplating a Fe-C alloy film onto an optical fiber core within the sensing region [18]. The research mentioned above needs extra packaging structures or sensing materials to transfer corrosion related parameters to fiber optic and make sensitive parameters detectible, which will inevitably affect durability and stability of corrosion sensors.

Distributed fiber optical Brillouin sensing is a cutting-edge technique in the field of structural health monitoring for infrastructures. Because of its advantages, such as distributed measurement ability, corrosion resistance, good durability, *etc.*, it has become a hot field of scientific research around the world. In recent years, its widespread applications in field works have made important contributions to the safe operation of structures. The Brillouin Optical Time Domain Reflectometer/Analysor (BOTDR/A), which is based on the propagation of a train of incident pulses and Brillouin back-scattering that occurs when light is transmitted through an optical fiber [19–22], can conduct continuous monitoring of the temperature and strain distributed over long distances [23,24]. The successful applications in different areas show that fiber optical Brillouin sensing technique offers a bright prospect owing to its distinct capability to monitor temperature and strain distribution along the sensing fiber optic [25].

Because of the long durability and electromagnetic interference immunity of the optical Brillouin sensing technique, the development fiber optical Brillouin corrosion sensors would be promising if proper sensing packaging methods can be found. The distributed strain sensing ability offers the opportunity to use the fiber optic coil winding method to directly measure the expansion strain caused by corrosion. The sensing packaging structure will be simple and no extra sensing materials and no complex packaging structure is needed. Based on fiber optical Brillouin technique, we propose a novel kind of fiber optic coil winding method to monitor the steel rebar corrosion expansion in reinforced concrete structures. Three kinds of novel Brillouin corrosion expansion sensors were designed and tested in this paper, in order to monitor corrosion development during long term service life of steel reinforced concrete structures.

## 2. Basic Principle of the Fiber Optical Brillouin Sensing Technique

The fiber optical Brillouin sensing technique is based on the Brillouin scattering phenomena. Fiber optics are utilized both as sensing elements and signal transmission media. When a short light pulse is launched into and transmitted along the fiber, the frequency of the Brillouin backscattering light can be measured at the same end. The time interval between sending the pulse and arrival of the backscattered light provides the spatial information, while the frequency of the backscattering light provides information of the temperature or strain distributed along the fiber optic. The backscattered Brillouin frequency is shifted from the incident light frequency because of the temperature variation and strain variation along the optic fiber. The Brillouin frequency shift is determined by the following equation:

(2.1) $$f_{B}=2{\mathrm{nv}}_{a}/\lambda$$

where *f_B_* is Brillouin frequency shift, *n* is refractive index of fiber, *v_a_* is the acoustic wave velocity and λ is wavelength of incident light.

The relationship between the Brillouin frequency shift at a certain location along the fiber and its corresponding temperature and strain change can be described as the following equation:

(2.2) $$f_{B}=k_{f\varepsilon }\Delta \varepsilon +k_{\mathrm{ft}}\Delta T+C$$

where Δ*T* is temperature change, *k_ft_* is temperature coefficient, *k_fε_* is strain coefficient of Brillouin frequency shift, Δε is strain change and *C* is a constant.

In the case of pure strain variations, there will be no temperature variations, so the relation can be simplified as:

(2.3) $$f_{B}=k_{f\varepsilon }\Delta \varepsilon +C$$

As a result, by measuring the Brillouin frequency shift we have access to the local temperature and strain conditions along the fiber optic.

## 3. Experiments

### 3.1. Strain Calibration Experiment

**Figure 2.**
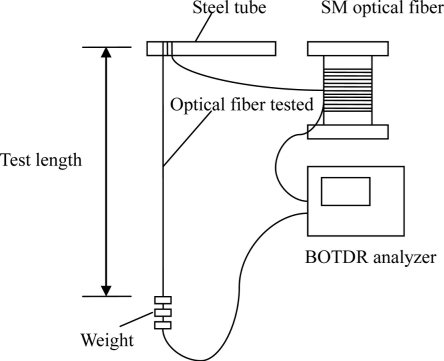
Setup of the strain calibration experiments.

Single mode fiber optics with sensing lengths of 1 m and 2 m were studied, and strain calibration experiments were designed and conducted to obtain the strain coefficients for monitoring corrosion expansion strain. In the experiments, a steel tube with 5 cm of diameter was used to support fiber optics with sensing lengths of 1 m or 2 m, respectively, as Figure 2 shows.

Weight was loaded and unloaded using weights ranging from 0 to 200 g, in increments of 50 g. Two load cycles were conducted for each kind of test length. Spatial resolution of 1 m, and a sampling interval of 0.41 m was used for every experiment. The Brillouin frequency was measured using BOTDA analyzer in every load step.

Diameter of fiber optic is 0.125 mm, and Young’s modulus is 0.72 × 105 N/mm^2^. According to elastic theory, there should be uniform strain distribution along the test length of fiber. When the weight of 50 g is loaded at the end of fiber, the strain change along the fiber is 558 με.

### 3.2. Design Principle of Brillouin Corrosion Expansion Sensors

**Figure 3.**
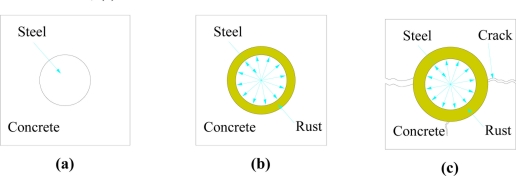
The corrosion process in the reinforced concrete; **(a)** without corrosion; **(b)** little amount of corrosion; **(c)** corrosion made concrete broken.

When corrosion happens, the volume of the steel rebar will increasingly expand due to the rust product accumulation on the surface of the steel rebar. Rust depth is reported as two to six times of the volume of steel lost during the corrosion process [1,2], as shown in Figure 3.

**Figure 4.**
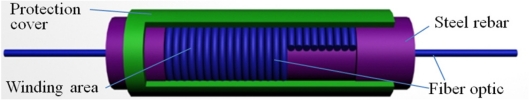
Basic packaging structure of the Brillouin corrosion expansion sensor.

Therefore, we wind the single mode fiber optic around the polished steel rebar into a fiber optic coil, with a little pretension, as Figure 4 shows. Then the fiber optic will be stretched when the volume of the steel rebar expands due to corrosion. Meanwhile, the tension strain change of the fiber optic coil in corrosion area can be monitored using the BOTDR/A analyzer, thus the corrosion process in the steel reinforced concrete structure can be monitored. Three kinds of Brillouin corrosion expansion sensors: BCES-I, BCES-II, BCES-III, were designed based on this principle. Different detailed packaging schemes were proposed for the three kinds of sensors to test the sensitivity for corrosion expansion monitoring. The single mode fiber optic used is Corning G.657A/B, with a minimum bending curve radius of 7.5 mm, which will keep optical losses at a very low level after dozens of fiber optic coils ares winded.

### 3.3. Design of BCES-I

**Figure 5.**
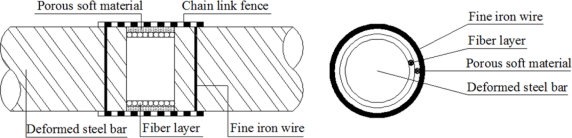
Packaging structure of BCES-I.

The design scheme of BCES-I is illustrated in Figure 5 and the corresponding sensor packaging method follows the steps described below: (1) polish the surface of a 20 mm-length part of a steel rebar with diameter of 22 mm (Φ 22), after which the diameter in polishing area decreased to 18.5 mm; (2) wind about 50 turns of single mode fiber optic into coils and guarantee the close contact of the fiber optic and steel rebar. The total winding length of the fiber optic is about 3.0 m; (3) fix both ends of the fiber on the rebar with epoxy glue, and draw out the fiber optic along the steel rebar; (4) put one layer of porous soft material outside the optical fiber to prevent damage to the fiber optic coil winding; (5) add chain link fence outside the porous soft material layer to resist impacts and the vibration effects of casting concrete; (6) fix the chain link fence firmly on the deformed steel rebar with fine iron wire.

The single mode fiber optic was wound around the polished steel rebar directly. There was nothing between the fiber optic and corrosion rust; so any corrosion expansion caused by rust accumulation will be detected by the fiber optic coil winding. Porous soft material and chain link fence was not only used to protect the fiber optic, but also to make the penetration of chlorides more easier than with concrete material alone. The BCES-I sensor was designed to detect the steel rebar corrosion expansion at early stage.

### 3.4. Design of BCES-II

**Figure 6.**
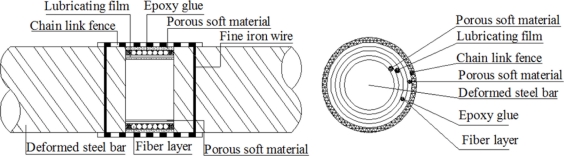
Packaging structure of BCES-II.

The design scheme of the BCES-II unit illustrated in Figure 6 makes the following changes based on BCES-I: (1) a porous soft material was added on the surface of the steel rebar and then waterproof lubricating film and fiber optic were wound; (2) the sensing optical fiber was coated with epoxy glue so that the optical fiber can deform in a coordinated fashion as a whole.

Single mode fiber optic was wound around the lubricating film layer. Corrosion expansion will act on the lubricating film layer first, and then on the fiber optic coil windings. Epoxy glue was used to provide better protection of the fiber optic. Because of the existence of lubricating film, the expansion strain along the winded fiber optic will be more even. Sensor BCES-II was designed to detect the steel rebar corrosion over a relatively big measurement range.

### 3.5. Design of BCES-III

**Figure 7.**
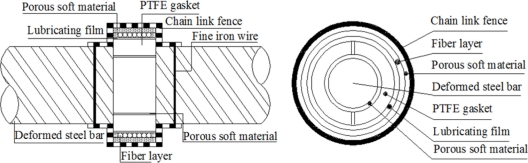
Packaging structure of BCES-III.

The design scheme of BCES-III is illustrated in Figure 7. It incorporates the following changes compared to BCES-I: (1) porous soft material was added on the polished steel rebar plus two polytetra-fluoroethylene (PTFE) gaskets whose internal surfaces closely contact the porous material layer; (2) thin lubricating film is wound on the PTFE gaskets, so as to minimize the stress accumulation generated in the contact area of the optical fiber and PTFE gaskets and thus guarantee compatible deformation of the sensing fiber after corrosion and expansion of the steel rebar; (3) the sensing fiber optic is wound around the lubricant film and the fiber coated with epoxy glue so that the fiber deforms uniformly as a whole.

Single mode fiber optic was wound around the lubricating film layer on the outside of PTFE gaskets. Corrosion expansion will act successively on the porous soft material layer, PTFE gaskets, lubricating film layer and finally on fiber optic coil windings. The BCES-III sensor was designed to detect the steel rebar corrosion expansion over a relatively big measurement range.

### 3.6. Accelerating Corrosion Experiment System

**Figure 8.**
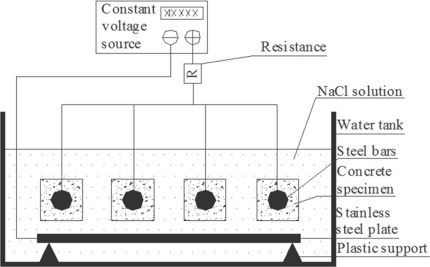
Schematic diagram of the electrochemical corrosion acceleration experiment system.

In order to study the sensitivity of the developed Brillouin corrosion expansion sensors, an electrochemical corrosion acceleration experiment system, shown in Figure 8, was built to accelerate the steel rebar corrosion process in concrete specimens. In the system, the steel rebar was the anode and the stainless steel plate was the cathode. When electric current was applied, the circuit between the steel rebar and the plate was conducted by Cl^−^, which will continuously transport Cl^−^ ions in the solution to the surface of the rebar. Cl^−^ is one of the most important factors influencing the corrosion of the steel rebar, and therefore this kind of experimental setup can accelerate the rate of corrosion enormously. During the experiment, the mass concentration of the solution of NaCl is 5.0%, and a constant voltage or constant current source was applied.

**Figure 9.**
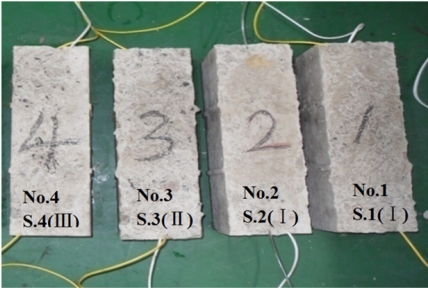
Concrete specimens with embedded sensors.

Before the experiment, the corrosion sensors were embedded into concrete specimens by concrete casting, and concrete specimens with embedded BCES-I sensors [sensor names: S.1 (I), S.2 (I)] were numbered specimens No. 1 and No. 2, respectively. The specimen with BCES-II [sensor name: S.3 (II)] was numbered No. 3, and the specimen with BCES-III [sensor name: S.4 (III)] was numbered No.4, as shown in Figure 9; the size of all concrete specimens was 100 mm × 100 mm × 300 mm. Concrete ultimate strength was 30 MPa. The cement used was P.O 32.5, and the concrete mass mix ratio was cement-water-sand-aggregate = 1:0.53:1.64:3.49. Only one sensor was embedded into each concrete specimen, placed along the axis of the specimen. After casting, the specimens were steam cured for 7 days, and then they were kept at room temperature at 22 °C for 3 days before they were tested.

The BOTDA analyzer used in the experiments is a DiTest STA202-C instrument manufactured by the Omnisens Company. It was used to collect monitoring data during the experiments, whose sampling interval was 0.41 m, spatial resolution 1.0 m, and strain resolution is 2 με. The BOTDA analyzer was set up to automatically scan the fiber optic circuit every one hour during the experiments.

**Figure 10.**
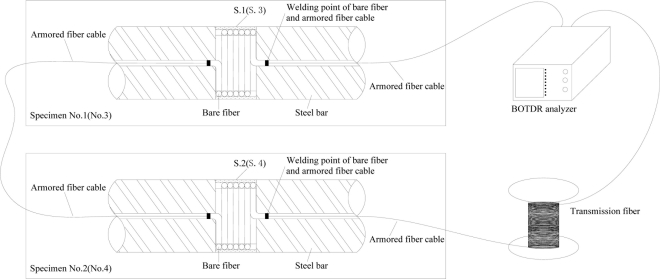
Fiber optic circuit of the corrosion monitoring experiments.

The experiments were divided into two parts and the fiber optic circuit is shown in Figure 10. Experiment one involved conducting the corrosion monitoring experiment of specimens No. 1 and No. 2. In the experiment, the fiber optics of S.1 (I) and S.2 (I) were connected together into one fiber optic circuit, and therefore quasi-distributed corrosion monitoring was conducted. A constant voltage source device was applied to supply electrical current. Voltage between the plate and the end of each specimen is 31.5 V. Experiment two was with specimens No. 3 and No. 4. Similarly, S.3 (II) and S.4 (III) were connected together into one fiber optic circuit. But in this case a constant current source was used, and the currents through the two specimens were 0.17 and 0.20 A, respectively.

## 4. Experimental Results and Discussion

### 4.1. Strain Calibration Experiment

**Figure 11.**
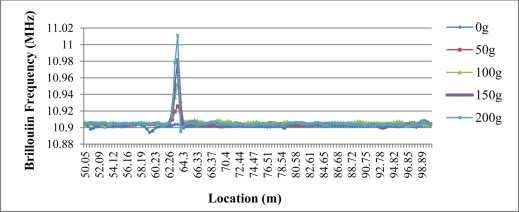
Brillouin frequency results of Load cycle 1 with sensing length of 1 m.

In the fiber optic circuit, only the sensing fiber optic with length of 1 m and 2 m is under extension load. There is signal change of the Brillouin frequency in the sensing fiber area, and no apparent signal change is found in any other part of fiber optic circuit, as Figure 11 shows. The signal peak grows gradually as loads from 0 g to 200 g were added in increments of 50 g. The maximum value at each signal peak is used to calibrate the strain coefficient.

**Figure 12.**
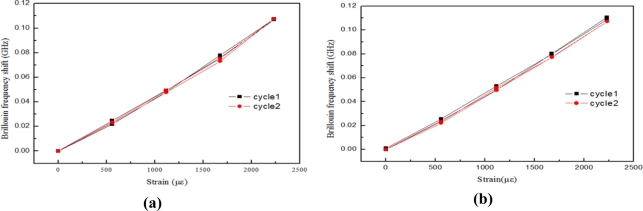
**(a)** Strain sensitivity result of sensor with length of 1 m; **(b)** Strain sensitivity result of sensor with length of 2 m.

There were very good linear relationships between strain and Brillouin frequency, when the test lengths are 1 m and 2 m, as Figure 12 shows. Also, good linear relationships between strain loaded and Brillouin frequency are found. An average strain coefficient of 0.048 MHz/με was obtained.

The strain calibration experimental results show that single mode fiber optics with sensing lengths of 1 m and 2 m can serve as strain sensors. The strain coefficients obtained with the two kinds of length show very good agreement. There is very good linearity of the results with the BOTDA analyzer using the spatial resolution of 1 m and sampling interval of 0.41 m.

### 4.2. Result Analysis of BCES-I

The electrochemical corrosion acceleration experiment was conducted on the two specimens No. 1 and No. 2 continuously until the signal of sensing fiber of S.1 (I) became so weak that the BOTDA analyzer could not detect it. Experiment 1 lasted for 35 h. A large crack caused by corrosion was found on surface of specimen No. 1 after the experiment.

**Figure 13.**
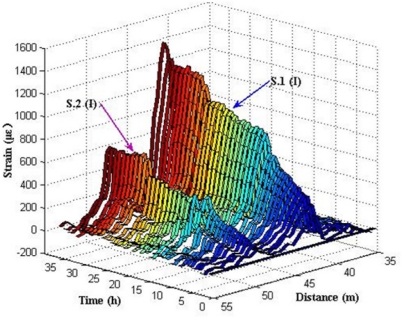
The expansion strain results of S.1 (I) and S.2 (I).

Figure 13 shows the relationship between distances, time and expansion strain measured by BOTDA analyzer.

In Figure 13, there are two expansion strain peaks at the distance 41–42 m and 48–49 m, where the fiber optic coil windings of sensor S.1 (I) and S.2 (I) are located in the fiber optic circuit of the experimental system. Results reveal that S.1 (I) and S.2 (I) can monitor the strain distribution in the fiber optic coil winding area caused by the steel rebar corrosion expansion in the specimens. No apparent strain change was detected in other locations of the fiber optic. Expansion strains increase steadily over time, which illustrates that the degree of corrosion grows gradually over time.

**Figure 14.**
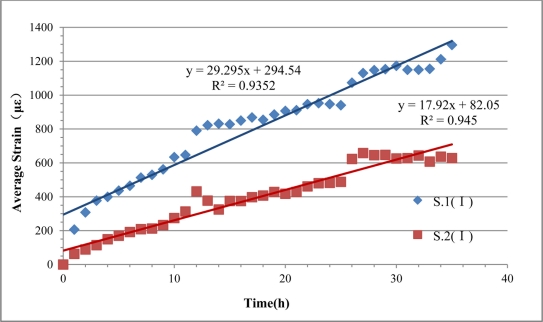
The average strains of sensor S.1 (I) and S.2 (I).

Furthermore, the average strains of the fiber optic coil windings of S.1 (I) and S.2 (I) and the straight-line fitting results were obtained, as shown in Figure 14. Because the voltage and the mass concentration of NaCl solution were constant and ignoring the non-uniform distribution of Cl^−^ in the solution, steel corrosion expansion will increase linearly. In Figure 14, the R^2^ values (square of correlation coefficient) of the corresponding fitted lines were 0.935 and 0.945, respectively, which shows that the average strain curves had good linearity during the corrosion monitoring experiment. The BCES-I monitoring results perfectly reflect the increasing trend of the linear development of steel corrosion, which indicates that this kind of sensor can monitor the real progress of steel corrosion, effectively.

According to the electrochemical method theory, the current is directly related to the rate of corrosion of steel rebars. The resistance of concrete will change a little in the experiment, so if the voltage is constant, the current will change during the experiment, and then corrosion speed of steel bars will change a little accordingly. Theoretically, the fitted lines should go through the origin of coordinates, however, as can be seen in Figure 14, neither of the fitted lines does, which is due to the reason that the unstable current under constant voltage in the initial phase brings about a surge in the steel corrosion rate. It can also be seen that average strains of sensing fibers in S.1 (I) and S.2 (I) increase over time, and the maximum strains reach 1,297 με and 658 με, separately.

**Figure 15.**
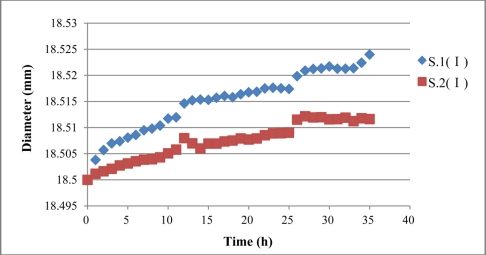
Steel rebar diameter monitored of sensor S.1 (I) and S.2 (I).

With the assumption of even corrosion during the experiment, there will be one layer of rust outside of the steel rebar with uniform thickness and also a linear relationship between circumference and diameter of the steel rebar with corrosion. The diameter of steel rebar with corrosion in sensor S.1 (I) and S.2 (I) can be calculated according to the average expansion strain result, as shown in Figure 15. Results show that diameter of sensor S.1 (I) increases to 18.524 mm, with a 0.024 mm increment compared to its initial status. The diameter of sensor S.2 (I) increases to 18.512 mm, with a 0.012 mm increment.

**Figure 16.**
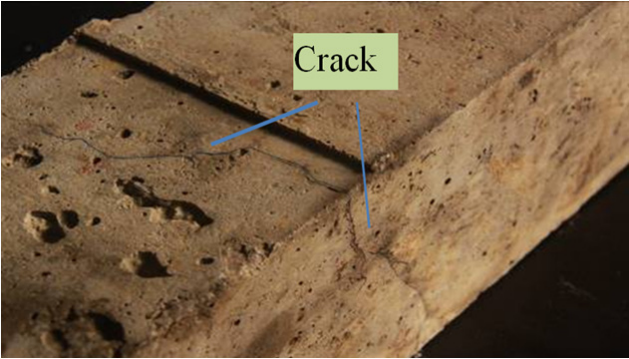
The crack on specimen No.1.

After the experiment, an obvious crack was found across three planes on the surface of specimen No.1, as Figure 16 shows. The crack ran from the bottom to the side plane and its length reaches 68 mm at the side plane. For practical applications, in most cases a crack with this length would severely threaten the safety of the structure. After the experiment, no apparent crack appears on the surface of specimen No. 2. Crack observation results showed good agreement with the expansion strain and diameter results.

**Figure 17.**
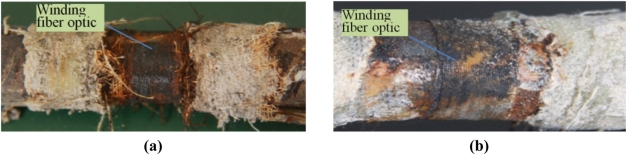
**(a)** The steel corrosion of S.1 (I) in the specimen No. 1; **(b)** The steel corrosion of S.2 (I) in the specimen No. 2.

The steel corrosion in specimen No.1 and No.2 after experiment one is shown in Figure 17. There is obvious corrosion rust distributed between the steel rebar and fiber optic coil windings.

Results of experiment one show that the advantages of BCES-I in the monitoring of steel rebar corrosion expansion can be summarized as follows:

1. The high sensitivity of BCES-I guarantees that the corrosion expansion strain of steel rebar can be monitored accurately and effectively. The corrosion signals were detected even within an hour after the experiment started, which reveals that early corrosion of steel rebars can be monitored effectively by this kind of sensor.
2. The steel corrosion expansion monitoring range of BCES-I is quite wide, from the beginning of corrosion to the appearance of large cracks in the specimen, which can satisfy the need of practical engineering applications.
3. BCES-I has good linearity and, it can carry out the real-time, quasi-distributed and quantitative steel corrosion monitoring.

The following disadvantages of BCES-I in the steel rebar corrosion expansion monitoring can be mentioned:

1. The sensing fiber optic coil winding of BCES-I has direct contact with the surface of the steel rebar, which leads to the fact that fiber optic is vulnerable when corrosion rust accumulates and large cracks of concrete appear; therefore, the sensor is not applicable to the condition of serious corrosion.
2. When the temperature varies greatly, the measured results are influenced a lot, and thus it is not applicable to environments with great variations of temperature, and some form of temperature compensation technique should be considered in future work. The improvement plan of BCES-I is to develop temperature compensation so that it can be applied in a temperature-varying environment.

### 4.3. Results Analysis of BCES-II

**Figure 18.**
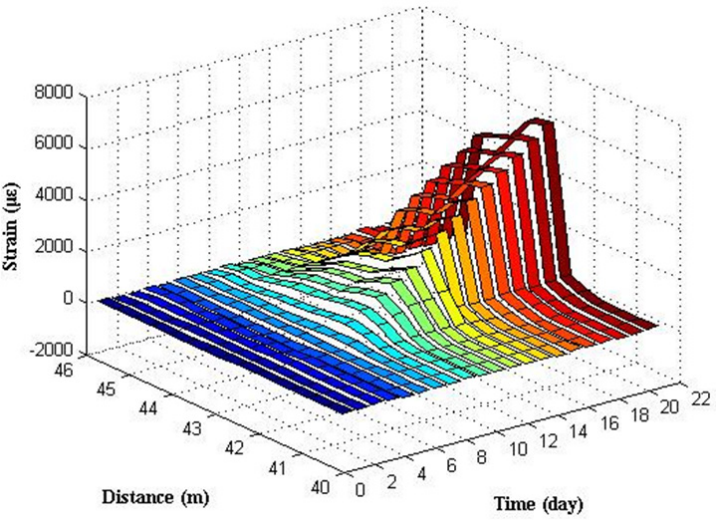
Corrosion expansion strain results of S.3 (II) in experiment two.

Electrochemical corrosion accelerating experiment two was conducted on specimens No. 3 and No. 4 continuously until the signal of sensing fiber optic became so weak that the BOTDA analyzer cannot detect it. The experiment lasted for 502 h (more than 20 days), which resulted in serious corrosion of specimens. Large cracks were found in two specimens. Figure 18 shows the relationship between distances, time and expansion strain of S.3 (II) measured by the BOTDA analyzer.

**Figure 19.**
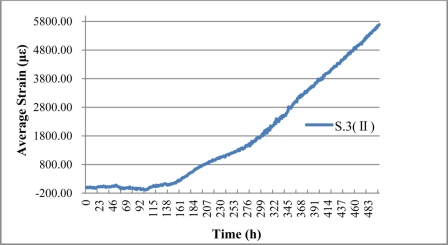
The average expansion strain result of S.3 (II).

In Figure 18, there clearly exists one strain peak in the fiber optic coil winding area, whose location in the fiber optic circuit is from 42.5 to 43.5 m. Result reveals that S.3 (II) can monitor the steel rebar corrosion expansion strain in the specimen. Because of the presence of a porous soft material layer and lubricating film between steel rebar surface and fiber optic coil winding, the expansion strain distribution along the sensing fiber became more even, and the peak became a little flat in comparison with BCES-I. No strain variation was measured along the fiber optic which is not located in the winding area. Expansion strain increased over time, which illustrates that the degree of corrosion grows gradually over time. Average expansion strain in the fiber optic coil winding area of S.3 (II) was calculated, as shown in Figure 19.

There is an evident growing trend over time, which reaches the maximum strain of 5,690 με at the 502th hour. BCES-II expands the corrosion monitoring range to about four times more than BCES-I. The reason is mainly attributable to the soft porous material and lubricating film located between the steel rebar surface and the wound fiber optic, which isolates the sensing fiber from the surface of the steel rebar, and reduce the micro bending caused by corrosion rust accumulation. Simultaneously, a small space in the porous material and tiny gaps between packaging layers will reduce the sensitivity of BCES-II, as BCES-□ only detected steel corrosion expansion after about 100 h, and then expansion strain increases linearly. The delay of corrosion expansion monitoring is also partially due to the epoxy glue’s protection from corrosion.

**Figure 20.**
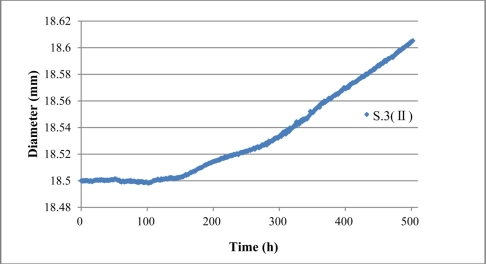
Monitored diameter of sensor S.3 (II).

With the assumption of even corrosion during the experiment, the diameter of the steel rebar in sensor S.3 (II) was calculated according to the average strain result, as shown in Figure 20. Results show that the diameter of sensor S.3 (II) increases to 18.605 mm, with a 0.105 mm increment over the initial status.

**Figure 21.**
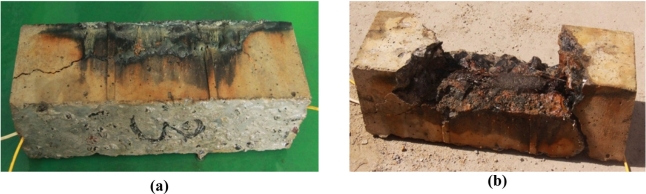
**(a)** the large cracks of specimen No.3; **(b)** Corrosion of the steel bar in specimen No.3.

After the experiment, obvious cracks across five planes appeared on the surface of specimen No. 3. The largest crack width along the length of the specimen is more than 3 mm on the bottom plane, and the cracks caused by corrosion have severely affected the specimen No. 3, from which it can be indicated that the measurement range of BCES-II is quite wide, and thus it can be applied to monitor serious steel corrosion in the concrete, as shown in Figure 21. The condition of the steel rebar after corrosion in specimen No. 3 is also severe.

From the analysis of the results of experiment two, the conclusions of the assessment and analysis of BCES-II are the following: compared with BCES-I, the relative low sensitivity of BCES-II makes it useless to accurately monitor the early corrosion of steel bars. However, the corrosion monitoring range of BCES-II is quite wide, and maximum expansion strain of steel rebar monitored reaches 5,690 με. Thus, it can be applied to monitor serious steel corrosion conditions in the concrete. The advantages of BCES-II are its big monitoring range, small packaging size, and its disadvantages are that its porous material make the sensor less sensitive compared to BCES-I.

Analysis of problems: the high porosity of the porous material layer of the BCES-II leads to the fact that expanding rust after corrosion must fill up the pores of the porous material layer before causing the expansion strain of the sensing fiber. Thus when the steel rebar corrosion is not serious enough, the sensor cannot detect any corrosion signal. At the same time, the sensing fiber does not have direct contact with the steel rebar under the protection of the porous material layer; thereby it is not easily damaged during the corrosion process. The improvement plan of the BCES-II is to reduce the thickness of the porous material layer so as to increase its sensitivity of steel corrosion monitoring, or to find some other kind of packaging materials with better compatibility with concrete materials. Meanwhile, it is better to apply the sensor to monitoring serious corrosion conditions.

### 4.4. Results Analysis of BCES-III

**Figure 22.**
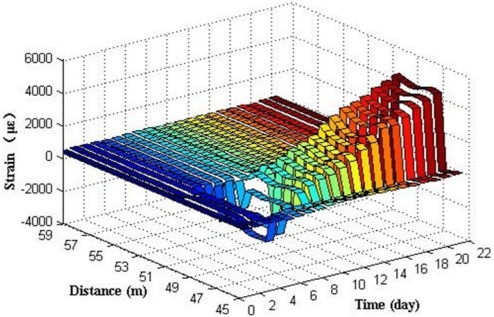
Corrosion strain results of S.4 (III).

In Figure 22, there clearly exists one expansion strain peak at the fiber optic coil winding area, whose location in the fiber optic circuit is from 48.0 to 49.0 m. The results reveal that S.4 (III) can monitor the steel rebar corrosion expansion strain in the specimen. Because of the presence of a porous soft material layer, lubricating film and PTFE gaskets between the steel rebar surface and fiber optic coil winding, the expansion strain distribution along the sensing fiber became more even, and the peak became more flat with in comparison to BCES-II.

**Figure 23.**
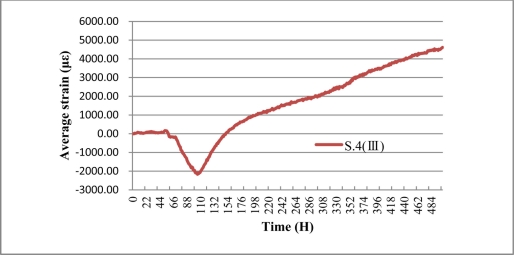
Average strain monitored of sensor S.4 (III).

The average expansion strain of S.4 (III) was calculated, as shown in Figure 23. The result shows that two phases of corrosion occur in the S.4 (III), the first one reduced the fiber strain gradually while the second one does the opposite. The main reasons for this can be justified as follows: in the first phase, the rust product fills the pores of the porous material layer and the diameter of the steel bar decreased. Then, under the action of the initial pretension applied on the fiber optic coil winding during the sensor packaging procedure, the two PTFE gaskets move relatively to be closer to each other, until the porosity was filled up. Accordingly, the strain of the fiber decreases gradually.

The second phase: during this phase, the rust product is left on the surface of the steel rebar. Under the action of rust expansive pressure, the two PTFE gaskets move relatively to be away from each other. Accordingly, the strain of the fiber increases gradually. Figure 23 shows that the average strain of S.4 (III), which shows a visible growing trend after a decline at the beginning stage, and reaches the peak of 4,610.7 με and the bottom of −2,126.7 με. A maximum expansion strain of 6,738 με was monitored. The delay of corrosion expansion monitoring is also partially due to the epoxy glue’s protection from corrosion.

Therefore, BCES-III expands the corrosion monitoring range greatly comparing to BCES-I, which mainly attribute to the PTFE gaskets and soft porous material that isolate the sensing fiber from the surface of steel rebars. Simultaneously, there exist the similar problem with BCES-II that the sensitivity of BCES-III declines.

**Figure 24.**
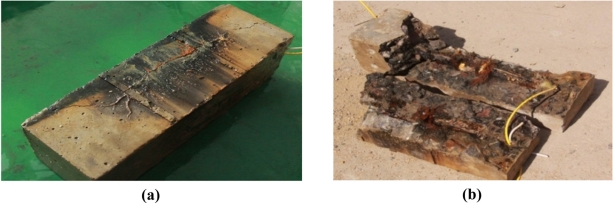
**(a)** The large cracks of the specimen No.4; **(b)** Corrosion of the steel rebar in specimen No.4.

After the experiment, obvious cracks across four planes were found on the surface of specimen No. 4. The largest crack width is more than 2.5 mm on the bottom plane, and the cracks severely damaged the specimen, as shown in Figure 24, from which it can be indicated that the measuring range of BCES-III is enormously wide, and thus it can be applied to monitor serious steel corrosion in concrete. The corrosion conditions of the steel rebar in this specimen were the most severe among all four specimens.

From the results mentioned above, the comprehensive assessment and analysis of BCES-III can be summarized in the following: compared with BCES-I, the relative low sensitivity of BCES-III makes it useless to accurately monitor the early corrosion of steel bars, making it similar to BCES-II. The corrosion monitoring range of BCES-III is enormously wide. Thus, it can be applied to monitor more serious steel corrosion in the concrete than BCES-II. The main advantage of BCES-III is its big monitoring range, however its disadvantages are that the PTFE gaskets make the sensor less sensitive compared to BCES-II, and there are tiny gaps between PTFE gaskets and the porous material. Also the packaging size is big, which will have influence on the local interface between steel rebar and concrete.

The improvement plan of BCES-III is to remove the porous material layer of the sensor or reduce its thickness so as to increase its sensitivity of the steel corrosion monitoring, or to replace the PTFE material with some other kind of material with better compatibility. Meanwhile, it is better to apply the sensor to monitor serious corrosion conditions.

### 4.5. Mass Loss Rate Estimate

#### 4.5.1. Derivation of the Evaluation Formula of Corrosion Mass Loss Rate

The corrosion mass loss rate evaluation formula was derived to estimate mass loss using the monitored expansion strain. The evaluation formula is only applicable to the assessment of monitoring results of BCES-I and BCES-II, and the following basic assumptions are made during the derivation:

1. Thickness of the fiber layer is rather small, approximately 1/72 of the diameter of the polished bar; therefore the fiber layer has little effect on the measurement results and thus its thickness is ignored during the derivation.
2. Thickness of the lubricating film and the porous material layer is quite small compared with that of fiber layer; hence it is ignored during the derivation.
3. All the rust produced during steel corrosion of the polished steel rebar remains inside the fiber layer (or lubricating film) without spilling out.
4. The cross sections of the steel rebar after corrosion are circular during the whole monitoring process with the presupposed uniform corrosion.
5. Temperature in the laboratory is constant; thus the measured results are not influenced by temperature.

Suppose the diameter of the polished rebar before corrosion is *D*, the diameter after corrosion is *D*_1_, the diameter including the rust layer after corrosion is *D*_2_, density of the steel bar is *ρ*, volume expansibility of the rust after corrosion is *η*, mass-loss rate of the steel bar after corrosion is *δ*, average tension strain along the optical fiber length in winding area is *ε*, then the derivation of the assessment formula of steel corrosion is as following:

The mass-loss rate of the steel bar at the monitoring position after corrosion is:

(4.1) $$\delta =\frac{\pi \left(D^{2}-D_{1}^{2}\right)\rho }{\pi D^{2}\rho }$$

It is simplified to:

(4.2) $$\delta =\frac{D^{2}-D_{1}^{2}}{D^{2}}$$

Equation (4.2) can be transformed into:

(4.3) $$\frac{D_{1}^{2}}{D^{2}}=1-\delta$$

The stain of the sensing fiber satisfies:

(4.4) $$\varepsilon =\frac{\pi \left(D_{2}-D\right)}{\pi D}$$

It can be simplified to:

(4.5) $$ɛ=\frac{D_{2}-D}{D}$$

The diameter of the steel bar satisfies the relationship:

(4.6) $$\eta \pi \left(D^{2}-D_{1}^{2}\right)=\pi \left(D_{2}^{2}-D_{1}^{2}\right)$$

It can be simplified to:

(4.7) $$\eta \left(D^{2}-D_{1}^{2}\right)=D_{2}^{2}-D_{1}^{2}$$

Equation (4.7) can be transformed into:

(4.8) $$\eta \frac{\left(D^{2}-D_{1}^{2}\right)}{D^{2}}=\frac{D_{2}^{2}-D^{2}}{D^{2}}-\frac{D_{1}^{2}}{D^{2}}+1$$

It can be further transformed into:

(4.9) $$\eta \frac{\left(D^{2}-D_{1}^{2}\right)}{D^{2}}=\frac{D_{2}-D}{D}\cdot \frac{D_{2}+D}{D}-\frac{D_{1}^{2}}{D^{2}}+1$$

Under the condition that the corrosion of the bar is not too severe, it’s safely assumed that:

(4.10) $$\frac{D_{2}+D}{D}\approx 2$$

Substituting Equations (4.2), (4.3), (4.5), (4.10) into Equation (4.9), the following equation can be obtained:

(4.11) $$\delta =\frac{2ɛ}{\eta -1}$$

**Figure 25.**
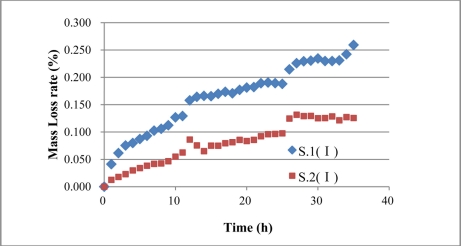
Mass loss rate results of S.1 (I) and S.2 (I).

**Figure 26.**
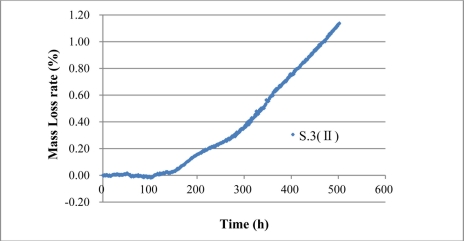
Mass loss rate results of S.3 (II).

Equation (4.11) is the mass loss rate evaluation formula of monitoring results of BCES-I and BCES-II. Assuming that volume expansion rate η is constant during the process of steel corrosion, and the value is 2, then the mass loss rates of the steel rebar of BCES-I and BCES-II over time can be obtained using Equation (4.11), as shown in Figures 25 and 26, which indicate that BCS-I and BCES-II can quantitatively assess mass loss rate through the evaluation formula. Mass loss rates of S.1 (I), S.2 (I) and S.3 (II) are 0.259%, 0.126% and 1.14%.

## 5. Conclusions and Future Work

The purpose of the research was to detect the corrosion expansion strain, and expansion diameter using the fiber optical Brillouin sensing technique which is durable and stable, and this will make the long term corrosion monitoring and evaluation possible. A novel kind of method named fiber optic coil winding method was proposed, discussed and tested to monitor corrosion expansion of steel rebar in steel reinforced concrete structures. Our strain calibration experimental result shows that there are good linear relationships between Brillouin frequency and strain applied. Average strain along the fiber optic coil winding can be monitored precisely using a BOTDA analyzer. Three kinds of fiber optical Brillouin expansion corrosion sensors suitable for different phases of steel corrosion in concrete structures with different sensitivity were developed in this paper, and their performance was tested in an electrochemical corrosion acceleration experiment system. BCES-I has a rather high sensitivity that it is able to detect signals of steel corrosion just at the beginning of the experiment, and average tension strain increased over time with good linearity, which reveals that early corrosion of steel rebar can be monitored accurately by this kind of sensor. The monitoring range of BCES-I is relative small, and it is thus suitable for detecting corrosion expansion at early stages. BCES-II has a relative low sensitivity compared with BCES-I, however the larger monitoring range with a maximum expansion strain of 5,690 με compensates for this to make it suitable for application in the serious corrosion situations. BCES-III has the lowest sensitivity but the largest monitoring range (maximum strain 6,738 με) of the three kinds of fiber optical Brillouin sensors. It can perform steel corrosion monitoring where there may exist an extremely corrosive environment. Furthermore, under the even corrosion assumption, diameter change caused by corrosion can be obtained through the average monitored expansion strain, which can be used to evaluate local corrosion damage directly. Finally, a mass loss rate evaluation formula was derived to help BCES-I and BCES-II estimate of mass loss rate. The studies show that distributed fiber optical Brillouin sensing technique using the fiber optic coil winding method can be used to effectively monitor and accurately evaluate quantitatively the steel corrosion in reinforced concrete structures.

There are still some work that needs further discussion and study in the future to make the corrosion monitoring more practical, such as temperature compensation technique, packaging structure optimization, and establishment of a distributed sensing network.
